# The current and future perspective of ChickenGTEx project and its applications in precision breeding

**DOI:** 10.1016/j.psj.2026.107132

**Published:** 2026-05-15

**Authors:** Lingzhao Fang, Haihan Zhang, Congjiao Sun, Xiangxiao Hu, Dominic Wright, Huaijun Zhou

**Affiliations:** a Center for Quantitative Genetics and Genomics, Aarhus University, Denmark; b College of Animal Science and Technology, Hunan Agricultural University, China; c Department of Animal Science and Technology, China Agricultural University, Beijing, China; d State Key Laboratory of Animal Biotech Breeding, College of Biological Sciences, China Agricultural University, Beijing, China; e Department of Animal Biosciences, Swedish University of Agricultural Sciences, Uppsala, Sweden; f Department of Animal Science, University of California, Davis, CA, United States

**Keywords:** ChickenGTEx, Integrative omics, Sex-dependent gene regulation, Extended laying cycle, Precision breeding

## Abstract

The Chicken Genotype–Tissue Expression (ChickenGTEx) project was established to systematically characterize the regulatory landscape of the chicken genome and to accelerate the translation of functional genomics into precision breeding. By integrating whole-genome sequencing with multi-tissue transcriptomic profiling, ChickenGTEx provides a comprehensive atlas of gene expression regulation across diverse tissues and physiological systems. Current findings demonstrate that complex production traits are governed by coordinated regulatory networks rather than isolated loci, with substantial contributions from tissue-specific gene expression, structural variation, and genotype-by-sex interactions. Sex-dependent regulatory effects further refine the genetic architecture of metabolic, immune, and reproductive traits, highlighting the importance of incorporating sex as a biological variable in genomic analyses. Application of integrative omics frameworks within elite layer populations has revealed multilayer regulatory mechanisms underlying extended laying performance, feed efficiency, metabolic health, and eggshell quality. By partitioning phenotypic variance into genetic, regulatory, and host–microbiome components, these approaches move beyond association-based mapping toward causal inference and biological interpretation. Importantly, validated regulatory loci identified through ChickenGTEx and related analyses provide actionable markers for genomic selection and rational targets for precision genome modification. Looking forward, continued expansion of regulatory atlases, incorporation of single-cell and longitudinal data in diverse environmental conditions, and integration of functional annotation into breeding pipelines will further enhance prediction accuracy and sustainable genetic improvement. The ChickenGTEx project thus represents a foundational platform bridging functional genomics and practical poultry breeding.

## Introduction

The chicken is the most widely produced livestock species globally and a cornerstone of sustainable animal protein production. Continuous genetic improvement has led to remarkable gains in growth rate, feed efficiency, egg production, and disease resistance over the past decades (Zuidhof et al., 2014). However, further progress in poultry breeding increasingly depends on a mechanistic understanding of how genetic variation influences complex and adaptive traits in a context-dependent manner across tissues, developmental stages, and environmental conditions. While whole-genome sequencing and genome-wide association studies (GWAS) have become routine in poultry research, translating genomic signals into actionable biological knowledge remains a major challenge.

A consistent outcome of GWAS in chickens is that most trait-associated variants reside in non-coding regions of the genome, often far from annotated genes (Maurano et al., 2012; Boyle et al., 2017). Although these loci are presumed to act through gene regulation, identifying the target genes, tissues, and molecular mechanisms involved is frequently difficult. This limitation hampers the interpretation of GWAS results, slows functional validation, and constrains the practical use of genomic information in precision breeding, disease resilience, and management strategies.

Expression quantitative trait locus (eQTL) mapping and related molecular QTL (molQTL) analyses provide a direct approach to bridge this gap by linking DNA sequence variation to molecular phenotypes such as gene expression, alternative splicing, and transcript processing. In humans, the Genotype-Tissue Expression (GTEx) project demonstrated that regulatory effects are often highly tissue-specific and context-dependent, fundamentally reshaping how complex traits are interpreted (GTEx Consortium, 2020). Comparable large-scale resources have until recently been lacking in poultry, limiting the ability to move from association to causation.

To address this need, the Farm Animal Genotype-Tissue Expression (FarmGTEx) initiative was established to systematically characterize genetic regulation of gene expression across tissues and biological contexts in major livestock species (Fang et al., 2025). Within this framework, the Chicken Genotype-Tissue Expression (ChickenGTEx) project represents the first comprehensive effort to map regulatory variants across the chicken genome using thousands of transcriptomes integrated with whole-genome sequence data from globally diverse populations. Results from the ChickenGTEx pilot phase have identified tens of thousands of molQTL across multiple tissues, demonstrating that regulatory variation is pervasive and plays a central role in shaping economically important traits in chickens (Guan et al., 2025).

Importantly for poultry science, many ChickenGTEx regulatory variants colocalize with GWAS signals for growth, carcass composition, reproduction, immunity, and metabolic traits. In numerous cases, the implicated regulatory genes are not the closest genes to the associated markers, highlighting why conventional annotation strategies can be misleading. Integration of ChickenGTEx with functional annotation resources generated by the FAANG initiative has further shown that regulatory variants are enriched in promoters, enhancers, and other active chromatin regions across chicken tissues (Pan et al., 2023), providing biological credibility to inferred variant–gene relationships.

This mini-symposium summarizes key findings from the ChickenGTEx pilot phase and discusses their implications for poultry genetics and genomics. We focus on how regulatory maps can improve interpretation of GWAS, enable identification of causal genes and pathways, and support the development of next-generation breeding strategies. We also outline future directions, including expansion to additional tissues, developmental stages, environmental challenges, and single-cell resolution, as well as the importance of international collaboration to build a comprehensive, context-specific regulatory atlas for chickens over the coming decade. Such efforts will strengthen the biological foundation of poultry breeding, enhance animal health and welfare, and reinforce the chicken’s role as both a production species and a model for vertebrate biology.

To establish a foundational framework for understanding regulatory mechanisms in chickens, the following section introduces the development and current achievements of the ChickenGTEx project, which provides the multi-tissue genomic infrastructure underlying subsequent analyses.

### The ChickenGTEx project: deciphering the regulatory landscape of the chicken genome

*Lingzhao Fang, Center for Quantitative Genetics and Genomics, Aarhus University, Denmark*

The Chicken Genotype–Tissue Expression (ChickenGTEx) project was established as part of the FarmGTEx initiative to generate a systematic map of genetic regulation of gene expression in chickens across tissues and biological contexts. Building on the overarching motivation outlined in the Introduction, this section focuses on the scope, analytical framework, and major findings of the ChickenGTEx pilot phase (Guan et al., 2025), which together provide a foundational resource for poultry functional genomics.

#### Project design and data integration

The ChickenGTEx pilot study integrates multiple large-scale genomic resources to enable comprehensive molecular quantitative trait locus (molQTL) mapping. Specifically, we assembled 7,015 high-quality bulk RNA-seq datasets spanning 28 tissues, single-cell transcriptomes from 127,598 cells, and whole-genome sequence (WGS) data from 2,869 individuals representing more than 100 globally distributed chicken breeds (Guan et al., 2025). This design enables joint interrogation of genetic regulation across transcriptional, post-transcriptional, tissue-specific, cellular, and population-level dimensions.

To maximize sample size and analytical power, single nucleotide polymorphisms (SNPs) were called directly from RNA-seq data and subsequently imputed using a global WGS reference panel. This strategy yielded approximately 1.5 million high-confidence variants and demonstrated statistical performance comparable to WGS-derived genotypes, establishing RNA-seq–based imputation as a scalable and cost-effective framework for large-scale molQTL mapping in chickens.

#### A multi-layer map of regulatory variation

Using this integrated dataset, ChickenGTEx systematically associated genetic variants with five classes of transcriptomic phenotypes: protein-coding gene expression, long non-coding RNA (lncRNA) expression, exon usage, alternative splicing, and 3′ untranslated region alternative polyadenylation (APA). In total, we detected significant genetic regulation for 13,983 protein-coding genes, 11,685 lncRNAs, 124,423 exons, 9,669 splicing events, and 8,798 APA sites (Guan et al., 2025).

Lead regulatory variants were strongly enriched near transcription start and transcription end sites, consistent with cis-regulatory control. More than 70 % of eGenes (genes associated with at least one eQTL) were regulated by multiple independent variants, revealing a modular and polygenic regulatory architecture. Genes harboring multiple independent eQTL displayed higher cis-heritability and lower evolutionary constraint compared with genes regulated by single or no detected eQTL.

To evaluate functional relevance, fine-mapped molQTL were significantly enriched in predicted regulatory DNA sequences, including promoters and enhancers defined by independent functional annotation resources in 23 chicken tissues (Pan et al., 2023). These regulatory effects were further supported by *in silico* mutagenesis using deep-learning models trained on regulatory genomics data and by allele-specific expression analyses, providing convergent evidence that the identified variants directly influence transcriptional regulation (Guan et al., 2025).

#### Distinct regulatory mechanisms across molecular phenotypes

ChickenGTEx revealed that different molecular phenotypes are often governed by partially distinct regulatory mechanisms. Colocalization analyses showed low to moderate sharing of causal variants between primary expression QTL (eQTL) and post-transcriptional QTL, including splicing QTL (sQTL) and APA QTL (Guan et al., 2025). Although all molQTL classes were enriched in promoter-like chromatin states and missense variants, the degree of overlap varied substantially among regulatory layers.

Integration with three-dimensional genome organization demonstrated that 20–60 % of molQTL–gene pairs were located within the same topologically associating domains, with strong enrichment at CTCF-associated chromatin loops. These findings highlight the importance of higher-order chromatin architecture in shaping regulatory variation in the chicken genome.

#### Tissue specificity and context-dependent regulation

Genetic regulation identified by ChickenGTEx was highly tissue- and context-specific. Blood formed a distinct regulatory outgroup, reflecting the presence of nucleated erythrocytes in birds, while embryonic tissues exhibited regulatory landscapes that differed markedly from adult tissues (Guan et al., 2025).

Beyond tissue-level effects, we mapped eQTL interacting with sex, transcription factor activity, and inferred cell-type composition. Cell-interaction eQTL (ci-eQTL) exhibited substantially lower sharing across cell types than across tissues, underscoring the fine-scale cellular specificity of avian gene regulation. For example, regulation of growth hormone expression in liver showed significant interaction with erythrocyte enrichment, illustrating how cellular context can modulate genetic effects.

#### Linking regulatory variation to complex traits

To connect regulatory variation with organism-level phenotypes, we integrated ChickenGTEx molQTL with genome-wide association study (GWAS) results for 39 complex traits using colocalization, Mendelian randomization, and transcriptome-wide association study (TWAS) approaches. Approximately 38 % of GWAS loci colocalized with at least one molQTL, and in 92 % of cases the implicated regulatory gene was not the nearest gene to the lead GWAS SNP (Guan et al., 2025).

One illustrative example is *KPNA3*, which was identified as a candidate gene for body weight gain through retina-specific eQTL, suggesting previously unrecognized links between sensory development and growth regulation. These results demonstrate how ChickenGTEx improves biological interpretation of GWAS signals and refines candidate gene prioritization in poultry.

#### Comparative and translational insights

Comparative analyses were conducted to evaluate the extent to which gene expression and regulatory architectures are conserved between chickens and mammals. Across chickens, humans, pigs, and cattle, orthologous genes exhibited broadly conserved tissue-specific expression patterns, with samples clustering primarily by tissue rather than by species. This finding indicates that core transcriptional programs underlying major physiological systems are deeply conserved across vertebrates (Guan et al., 2025).

In contrast, the genetic regulation of gene expression showed greater divergence. Cross-species comparisons of cis-heritability and lead eQTL effect sizes for orthologous genes revealed significant but generally weak correlations between chickens and mammals.

Integration of transcriptome-wide association studies (TWAS) enabled functional comparison of complex trait genetics across species. Significant cross-species correlations were identified for multiple traits, including shared regulatory signals linking chicken body weight with growth-related traits in pigs. These results demonstrate that comparative regulatory genomics can uncover conserved biological pathways while also highlighting species-specific regulatory mechanisms that must be considered when translating findings across taxa.

Together, these analyses position ChickenGTEx as a valuable comparative resource, supporting both poultry-focused genetic improvement and broader studies of vertebrate gene regulation.

#### Summary

The ChickenGTEx project provides the most comprehensive reference map of genetic regulation in birds currently available (https://chicken.farmgtex.org). By establishing direct links between genetic variants, regulatory mechanisms, and complex traits, this resource lays the groundwork for precision breeding, functional validation, and systems-level studies of avian biology.

Future expansions will incorporate pan-genome representations, structural variant QTL, life-stage–specific mapping, single-nucleus transcriptomics, and regulatory responses to environmental challenges such as nutrition, heat stress, and infectious disease. Together, these efforts will enable a dynamic, context-aware understanding of gene regulation in chickens and directly support next-generation poultry genetics and genomics research.

Building upon this multi-tissue regulatory atlas, the next contribution examines how genetic regulation differs between males and females, highlighting the importance of incorporating sex as a biological factor in functional genomic analyses.

### Sex-biased gene regulation across 30 chicken tissues

*Haihan Zhang, College of Animal Science and Technology, Hunan Agricultural University, China*

Sexual dimorphism is a defining biological feature of chickens and a foundational component of modern poultry production systems. In contrast to mammals, which rely on an XY sex-determination system, birds utilize a ZW system in which males are homogametic (ZZ) and females are heterogametic (ZW) (Graves, 2016). This chromosomal architecture influences gene dosage, epigenetic regulation, and transcriptional activity across tissues. Unlike mammals, birds exhibit incomplete dosage compensation of Z-linked genes, resulting in persistent sex differences in gene expression beyond the reproductive system (Graves, 2016). These genomic characteristics provide a biological framework in which sex-biased gene regulation is expected to be widespread and functionally important.

Sex bias in chickens encompasses not only differences in reproductive biology but also divergence in growth, metabolism, immune function, and behavior. The functional effect of a genetic variant may differ between sexes even when allele frequencies are identical, a phenomenon referred to as sex-biased or sex-dependent genetic regulation (Fallahshahroudi et al., 2025; Papanicolaou et al., 2025). Such effects can arise from differences in sex chromosome dosage, hormone-responsive transcription factor activity, chromatin accessibility, or sex-specific cellular composition. In poultry production populations, these mechanisms are particularly relevant because selection objectives differ substantially between males and females. Broiler breeder males are typically optimized for rapid growth, feed efficiency, and muscle accretion, whereas females, especially in layer and breeder female lines, are selected for reproductive performance, egg production persistence, lipid metabolism, and immune competence.

To better understand the genetic basis of this dimorphism, we recently established a sex-balanced multi-tissue regulatory resource, referred to as ChickenSexGTEx (Zhang, 2026). This cohort integrates whole-genome sequencing with approximately 8000 RNA-seq data collected across 32 primary tissues from uniformly reared male and female chickens at a synchronized developmental stage (90 days). By minimizing environmental and developmental confounders, this design provides a controlled physiological framework for the robust interrogation of genotype-by-sex (*G* × Sex) regulatory interactions that are often subtle and easily obscured in less controlled settings.

#### Widespread transcriptional dimorphism across tissues

Analysis across non-gonadal tissues revealed that sex-biased expression is pervasive, affecting a substantial fraction of the annotated genome in at least one tissue. The magnitude of dimorphism varies by tissue, with skeletal, immune, and neuroendocrine organs exhibiting particularly strong divergence, whereas several digestive tissues show comparatively modest differences. Although Z-linked genes contribute prominently to male-biased expression due to incomplete dosage compensation, the majority of sex-biased genes reside on autosomes, indicating that systemic and regulatory mechanisms extend well beyond sex chromosomes.

Functionally, male-biased expression patterns are enriched for musculoskeletal growth and structural pathways, whereas female-biased genes tend to involve lipid metabolism and immune-related processes. These patterns are consistent with sex-specific allocation of physiological resources and provide a molecular explanation for well-established phenotypic differences in growth rate, body composition, and immune responsiveness.

#### Sex-interacting regulatory variants represent a focused subset

Despite widespread differential expression, direct sex-dependent genetic regulation appears to be more selective. Using a *G* × Sex interaction framework, we identified several hundred regulatory variants whose effects differ between males and females. These variants influence a focused subset of genes and are often tissue-specific, with enrichment observed in endocrine and metabolic organs.

Importantly, the overlap between genes showing differential expression and those harboring sex-interacting eQTL is limited. This suggests that most transcriptional dimorphism arises from hormonal signaling and systemic physiological environments, whereas a smaller subset reflects true genotype-by-sex interactions. Some regulatory variants exhibit unequal effect sizes between sexes, and a smaller fraction display opposite allelic directions, consistent with theoretical models of sexual antagonism and resolution of sex-specific selective pressures.

#### Cellular composition as a contributor to apparent dimorphism

Because bulk transcriptomic profiles are influenced by cellular heterogeneity, we integrated complementary single-cell data to evaluate sex differences in tissue composition. Several immune and stromal cell populations exhibit sex-biased abundance across tissues. Adjustment for cell-type proportions reduces the magnitude of apparent sex-biased expression for many genes, indicating that a portion of transcriptomic dimorphism reflects compositional differences rather than intrinsic regulatory changes.

At the same time, cell-type–dependent regulatory effects are detectable, demonstrating that genetic variants may exert context-specific influence that depends on both tissue identity and cellular composition. These findings emphasize that sexual dimorphism emerges from the combined effects of chromosomal dosage, endocrine signaling, and cellular architecture.

#### Structural variation and higher-order phenotypes

In addition to single-nucleotide variants, structural variants (SV) contribute to tissue-specific regulatory landscapes and, in some cases, exhibit sex-dependent effects. Incorporating SV modestly increases the proportion of gene expression variance explained and highlights regulatory signals that would be missed by SNP-only analyses.

Integration of regulatory maps with metabolic phenotypes further indicates that a subset of sex-biased regulatory variants is associated with sexually dimorphic traits, including lipid metabolism and growth-related measures. These observations suggest that sex-dependent regulation contributes to higher-order physiological divergence and may influence economically important traits.

#### Implications for poultry genetics

Collectively, these findings support a multi-layered model of sexual dimorphism in chickens. Widespread transcriptional differences arise from sex chromosome dosage effects and systemic hormonal environments, while a more limited but biologically meaningful set of regulatory variants exhibits sex-dependent genetic effects. Cellular composition and structural variation further refine this architecture.

For poultry breeding, these results highlight that treating sex solely as a covariate may obscure biologically relevant regulatory interactions. Incorporating sex-dependent regulatory information into genomic analyses could improve interpretation of GWAS signals and support more precise breeding strategies tailored to sex-specific production objectives.

While sex-dependent regulatory effects represent one important layer of phenotypic variation, production traits are also shaped by dynamic physiological processes across the laying cycle. The following section focuses on integrative multi-omics analyses in elite layer populations to dissect the genetic regulation of extended laying performance.

### Genetic regulation of chicken production traits during an extended laying cycle based on integrative omics

*Congjiao Sun, Department of Animal Science and Technology, China Agricultural University, China*

For decades, the global layer industry has pursued higher efficiency, lower costs, and more sustainable production systems (Bist et al., 2024). Extending the laying cycle of hens has emerged as a core strategy to meet these demands. While traditional cycles typically last 72–80 weeks and yield approximately 320–360 eggs, extending production to 100 weeks offers clear economic and environmental advantages (van de Braak, 2025). Achieving 500 eggs in 100 weeks represents a new breeding benchmark for modern layer systems.

However, prolonged laying presents substantial biological challenges. Production traits, including egg mass, shell strength, and feed efficiency, decline during late lay (Molnár, 2017). Metabolic disorders become more prevalent, with increased risk of hepatic steatosis (Guo et al., 2023) and excessive abdominal fat deposition that compromises efficiency and carcass quality (Liu et al., 2024). These issues reflect cumulative physiological stress, disruption of lipid metabolism, and imbalances in endocrine regulation.

#### From association to mechanism: integrating multi-omics

A major bottleneck in improving late-lay performance lies in limited understanding of the regulatory mechanisms linking genotype to phenotype. Conventional GWAS identify loci associated with traits but rarely clarify how genetic variants act through regulatory networks across tissues.

To address this gap, we established an integrative omics framework in a large high-yield Rhode Island Red pure line population. Unlike multi-breed mapping efforts such as the FarmGTEx project, which emphasize cross-population regulatory architecture, our focus was mechanistic dissection within a single elite breeding population to enable direct translational application.

A total of 953 hens were followed to 100 weeks of age with comprehensive phenotyping, including reproductive traits, feed efficiency, carcass characteristics, biochemical indicators, abdominal fat deposition, and egg quality. Genome-wide genotyping and transcriptomic profiling were performed across ten key tissues spanning the hypothalamic–pituitary–ovarian axis, metabolic organs, intestine, and oviduct. Intestinal microbiome profiling was also incorporated to capture host–microbe interactions relevant to nutrient utilization and lipid metabolism.

#### Partitioning phenotypic variance across biological layers

To quantify the relative contributions of host genetics, gene regulation, and intestinal microbiota, we developed a heritability–regulatability–microbiability (h²–r_b_²–m²) framework. This model extends classical quantitative genetics by incorporating molecular regulatory and microbial effects into variance partitioning (Lan et al., 2025), and overcomes the limitations of traditional quantitative genetics models (which only consider genetic effects), and allows for the simultaneous evaluation of multiple causal factors. The microbiability (m²) of residual feed intake (RFI) in the duodenum and cecum was 0.24 and 0.21 (Wen et al., 2021), respectively, indicating that intestinal microbes play a crucial role in regulating feed efficiency. For yolk weight, the regulatability (rb²) of the pituitary gland and liver was the highest (0.25-0.40), highlighting the key regulatory role of these tissues.

Through eQTL mapping, we identified a significant tissue-specific regulatory pattern. The hypothalamus, pituitary gland, and liver exhibited strong and stable *cis*-regulatory effects, and the proportion of eGenes exceeded 50 % (50.37 %, 56.85 %, 50.84 % in the hypothalamus, pituitary gland and liver), and cis-heritability (h_cis_²) was 0.14-0.16. In contrast, reproductive tissues had much weaker *cis*-regulatory effects. The proportion of eGenes was only 26.08 % in the magnum and 30.51 % in the uterus, with h_cis_² of 0.06-0.07. This pattern was consistent across different molecular phenotypes, including transcripts, exons, and 3′ APA UTR. Besides, eGenes with multiple independent eQTL showed increased h_cis_² values.

#### Decoding causal mechanisms of key extended-lay traits

Using integrative analyses and causal inference approaches, we investigated five economically important traits: egg production persistence, feed efficiency, hepatic steatosis, abdominal fat deposition, and eggshell quality.

Egg production across an extended cycle is regulated by coordinated interactions between the hypothalamic–pituitary–ovarian axis and metabolic tissues. Both age-shared and age-specific regulatory loci were identified, supporting the concept that early-lay and late-lay productivity are partially governed by distinct molecular mechanisms.

Feed efficiency analysis identified a regulatory locus affecting expression of SUCLA2, a key enzyme in the tricarboxylic acid cycle (Zhang et al., 2024). Enhanced SUCLA2 expression was associated with improved energy metabolism, supporting sustained egg production without excessive fat accumulation.

For hepatic steatosis, a major regulatory locus influencing PEMT expression was detected (Sun et al., 2024). PEMT plays a central role in phosphatidylcholine synthesis and very-low-density lipoprotein (VLDL) assembly. Reduced PEMT activity impairs lipid export from the liver, predisposing hens to triglyceride accumulation during prolonged laying.

Abdominal fat deposition was found to be jointly influenced by host regulatory variants and intestinal microbial composition. We identified two eQTLs on chromosome 3 that were associated with abdominal fat weight (AFW) by affecting the expression of the CHST14 gene and the abundance of *Lactobacillus salivarius* in the duodenum (Lan et al., 2025), suggesting a coordinated host–microbe mechanism involving bile acid metabolism and enterohepatic lipid circulation.

Together, these findings demonstrate that extended laying traits are shaped by multi-layer regulatory interactions spanning endocrine signaling, hepatic lipid metabolism, intestinal function, and microbial ecology.

#### Translation into genomic breeding

Importantly, mechanistic discoveries were integrated into practical breeding strategies. In collaboration with industry partners, we developed a genomic selection platform incorporating validated regulatory markers. A custom genotyping chip was applied to large-scale performance testing and selection programs.

This integrative genomic breeding scheme contributed to the development of the “Jinfeng No.6″ commercial layer line, which achieves an average of approximately 500 eggs by 100 weeks of age, with prolonged high laying rate and stable eggshell quality. These results demonstrate that integrating molecular regulatory information with traditional crossbreeding can accelerate genetic progress in extended-cycle performance.

#### Summary

Extended laying represents a complex, time-dependent phenotype governed by coordinated regulation across endocrine, metabolic, intestinal, and reproductive systems. Integrative omics approaches enable dissection of causal pathways linking genetic variation to production traits, moving beyond association toward mechanism.

While broad regulatory atlases such as ChickenGTEx identify large-effect variants across diverse populations, focused analysis within elite breeding lines allows identification of both major and moderate-effect regulatory variants directly applicable to selection. Incorporating regulatory markers and host–microbiome interactions into genomic prediction models can enhance selection accuracy and support sustainable improvements in long-cycle productivity.

Beyond mechanistic insight, translating regulatory discoveries into practical breeding strategies is essential. The integration of genomic, transcriptomic, and microbial information provides actionable targets for genomic selection and precision genome modification.

### Mapping the genetic regulatory landscape of chicken growth traits using a chicken advanced intercross line and multi-omics data

*Xiaoxiang Hu, State Key Laboratory of Animal Biotech Breeding, College of Biological Sciences, China Agricultural University, China*

Growth traits are central targets of genetic improvement in poultry, yet the biological pathways linking genetic variation to growth performance remain highly complex due to the involvement of multiple tissues, developmental stages, and regulatory mechanisms. Although artificial selection has achieved substantial gains in growth rate and efficiency (Zuidhof et al., 2014), many loci underlying growth-related traits remain difficult to interpret because associated variants are frequently located in non-coding regions and exert small, context-dependent effects (Maurano et al., 2012; Boyle et al., 2017). This complexity necessitates high-resolution mapping populations and integrative analytical frameworks that combine genetic, regulatory, and functional genomic data.

Traditional animal genetic mapping has relied primarily on F2 populations; however, mapping resolution in such designs is constrained by extensive linkage disequilibrium, limiting the ability to localize causal variants for complex traits (Besnier et al., 2011; Sheng et al., 2013). Advanced intercross lines (AIL) and multi-parent advanced generation inter-cross (MAGIC) populations accumulate recombination over successive generations, substantially improving mapping precision and enabling finer dissection of quantitative trait loci (Gonzales et al., 2018; Yang et al., 2022). These considerations motivated the development of a long-term chicken AIL for high-resolution mapping of growth-related traits.

#### Development of a high-resolution advanced intercross line

In parallel, rapid advances in functional genomics resources for farm animals, including FarmGTEx and FAANG, have greatly expanded the availability of gene expression and regulatory annotation data in chickens (GTEx Consortium, 2020; Pan et al., 2023; Fang et al., 2025; Guan et al., 2025), providing a critical framework for linking fine-mapped loci to functional genes and regulatory mechanisms.

To improve mapping resolution beyond that achievable in conventional F2 populations, we established a long-term chicken advanced intercross line (AIL) derived from Huiyang Bearded chickens and Lingnan Yellow line A03, which differ markedly in body weight. This population was maintained through successive generations of random mating over 17 years (Wang et al., 2020a). Population genetic analyses showed stable nucleotide diversity, low inbreeding coefficients, and rapid decay of linkage disequilibrium across generations, indicating minimal genetic drift and progressive fragmentation of ancestral haplotypes. These features make the AIL a powerful resource for fine mapping of complex traits.

Using the F16 generation, we performed genome-wide association studies (GWAS) for 75 phenotypes spanning five major trait categories. Significant associations were identified for 43 traits, and fine mapping resolved 682 quantitative trait loci (QTL). Notably, QTL intervals decreased from an average of 1.77 Mb in the F2 generation to 238 kb in F16, demonstrating the substantial gain in mapping precision achieved through multigenerational recombination. Several previously characterized loci were recovered, including *HOXB8* for muffs and beards and *SOX10* for plumage pigmentation (Gunnarsson et al., 2011; Guo et al., 2016), validating the robustness of the approach.

#### Integrating regulatory genomics to identify functional variants

Despite improved mapping resolution, functional annotation revealed that most significant SNPs were located in intronic (55.55 %) or intergenic (17.17 %) regions, emphasizing the importance of regulatory mechanisms. To address this, we integrated multiple complementary resources, including the Global Chicken Reference Panel (GCRP), FAANG functional annotations, and gene expression regulation data from ChickenGTEx (Pan et al., 2023; Guan et al., 2025; Zhu et al., 2025b).

Across 446 analyzed QTL, 245 were supported by expression quantitative trait loci (eQTL), leading to the identification of 431 candidate functional genes. Integrative analyses revealed four major modes of variant-mediated regulation. These included (1) cis-regulatory variants directly influencing phenotypes, exemplified by *ST3GAL4*, where a small intestinal regulatory variant affected serum alkaline phosphatase levels; (2) long-range, tissue-specific regulation, such as a shank length QTL mediated by distinct genes across muscle, embryonic tissue, and intestine; (3) generation-specific QTL arising from allele frequency shifts, illustrated by an eviscerated weight locus near *NCAPG*; and (4) clustered multi-gene regulation, where physically linked genes across multiple tissues collectively influenced body weight, creating a pseudo–major-effect QTL consistent with an omnigenic model.

Haplotype analysis across divergent chicken lines further supported the functional relevance of this clustered architecture, revealing incomplete fixation of favorable haplotypes and suggesting continued potential for genetic improvement at these loci (Zhu et al., 2025a).

#### Conservation and divergence of growth-related regulation

To evaluate the evolutionary conservation of genetic mechanisms underlying growth traits, genes significantly associated with multiple chicken growth phenotypes were mapped to their orthologs in humans. More than 80 % of growth-associated chicken genes had identifiable human counterparts, and many of these orthologs have been implicated in growth, metabolism, or body composition–related traits in humans. This high degree of conservation at the gene level indicates that core biological pathways regulating growth are shared across vertebrates and reinforces the relevance of chickens as a comparative model for complex trait biology (Zhu et al., 2025a).

In contrast, conservation was markedly lower at the variant level. Only a small fraction of growth-associated SNPs could be directly mapped to the human genome, and their regulatory annotations often differed substantially between species. Furthermore, chicken growth-related genes exhibited shorter intronic and intergenic regions compared with their human orthologs, reflecting differences in genome architecture and regulatory landscapes (Zhu et al., 2025a). These observations suggest that, although the same genes frequently contribute to growth across species, the regulatory variants and genomic contexts through which they act have undergone substantial lineage-specific reorganization during vertebrate evolution.

Together, these results highlight a key distinction between conserved biological function and divergent regulatory implementation. For poultry genetics, this implies that functional gene discovery can benefit from comparative genomics, whereas identification of causal regulatory variants requires species-specific resources, including high-resolution mapping populations and chicken-focused functional genomics datasets such as ChickenGTEx (Pan et al., 2023; Guan et al., 2025).

#### Summary

This study demonstrates the value of integrating a long-term advanced intercross line with multi-omics data to resolve the regulatory architecture of chicken growth traits. By leveraging extensive recombination accumulated over multiple generations and combining genome-wide association analyses with functional annotation and gene expression regulation, we were able to move beyond locus identification toward precise variant–gene–phenotype relationships (Wang et al., 2020a; Zhu et al., 2025a).

The identification of multiple regulatory modes including cis-regulatory effects, long-range tissue-specific regulation, generation-dependent QTL, and clustered multi-gene architectures, highlights the complexity of growth trait regulation and underscores the limitations of interpreting GWAS results in isolation (Boyle et al., 2017; Zhu et al., 2025a). Importantly, the incomplete fixation of favorable haplotypes at several key loci suggests that substantial genetic potential for continued selection remains in current chicken populations.

Looking forward, further gains in resolution and biological insight will be achieved by incorporating multi-population GWAS designs to increase genetic diversity and robustness of association signals (Yang et al., 2022). Improved integration of transcriptomic datasets across tissues and developmental stages, together with emerging single-cell and single-nucleus approaches, will enable finer dissection of cell-type–specific regulatory mechanisms. Collectively, these advances will strengthen the biological foundation of poultry breeding and facilitate the development of more precise, mechanism-informed strategies for improving growth and related traits.

While genomic selection leverages naturally occurring variation, functional validation of key regulatory loci also opens opportunities to directly modify quantitative phenotypic variation through targeted transgenic and gene-editing technologies.

### Identifying loci for modifying large-scale quantitative phenotypic variation via transgenic technology

*Dominic Wright, Department of Animal Biosciences, Swedish University of Agricultural Sciences, Sweden*

The expanding regulatory landscape defined by the ChickenGTEx project provides not only mechanistic insight into complex trait architecture but also a strategic foundation for next-generation genetic improvement. By integrating multi-tissue expression profiling, causal inference, and multi-omics analyses, we can now prioritize regulatory loci that exert coordinated effects across growth, metabolic efficiency, reproductive persistence, and resilience traits. While traditional genomic selection capitalizes on existing allelic variation, the precise identification of functional regulatory elements opens new possibilities for targeted genome modification. Transgenic and gene-editing technologies offer complementary approaches to experimentally validate candidate loci and to explore the controlled modulation of quantitative phenotypic variation at scale. As regulatory genomics continues to mature, the integration of functional validation with precision genome engineering may enable more predictable and biologically informed improvement strategies, bridging discovery-driven genomics with transformative applications in poultry breeding.

With the advent of de-extinction, science is attempting some of the most extreme phenotypic alterations at the genomic level to date, and has certainly captured the public imagination (Shapiro, 2020). Typically (though not exclusively) the technology behind these initiatives involves taking the nearest living relative to the extinct species and then altering the phenotype to match that of the extinct species as much as possible (Turner et al., 2025). Depending on the degree of differences between the extant and extinct species, this can entail considerable alterations, not only morphological but also physiological and behavioural in nature. For example, in the case of the woolly mammoth being de-extincted from the elephant, traits ranging from the long double-coated fur, greater subcutaneous fat, smaller ears and limbs, enlarged and twisted tusks, and wider feet are all different (Kubiak, 1982). In another example, the extinct passenger pigeon’s closest living relative is the band-tailed pigeon (Murray et al., 2017). In this case, this involves some morphological traits (longer tail and iridescent pink breast patch), but also large behavioral differences (the passenger pigeon bred in large colonies, and lived in huge flocks of hundreds of thousands of birds (Blockstein, 2017)). Such extreme phenotypic modification is no small feat to perform, and asks the question if transgenic rewriting of the genome is actually feasible to induce such alterations in sufficient volume (Shapiro, 2017).

De-extinction via genome engineering is perhaps the most universally viable strategy to date, given the issues with somatic cell nuclear transfer (cloning) requiring a whole preserved cell, and selective back-breeding requiring a living relative still possessing the desired traits, as well as being very time intensive (Turner, Keyte, Pask and Shapiro, 2025). Whilst the technology to produce the actual genome engineering and gametes has increased, this still leaves the problem of how to identify the exact genomic regions that are required to insert to give rise to the altered phenotype. For example, is it even possible to take an extant animal and modify its phenotype to that of another, related, extant animal or even a sub-type (for example a domestic animal to the corresponding wild progenitor)? In this example, a much wider range of tools are available to identify the requisite loci. In particular, genetic crossings, genome wide association, and linkage mapping can identify Quantitative trait Loci (QTL) and single gene mutations or polymorphisms and are routinely used to try and identify such genetic loci segregating both within and between populations (Gallagher and Chen-Plotkin, 2018; Lynch and Walsh, 1998; Visscher et al., 2012). Although these tools are likely not available when attempting a full de-extinction project, such a test would be an important validation as an indicator of the viability of de-extinction projects in general. If it is not possible to take an extant species and engineer it to alter its phenotype into something different (ideally a different extant sub-population or sub-species), with all the additional tools we can bring to bear using such a species, how will it be possible to perform the same for an extinct species? Such an experiment would therefore serve as an important proof of principle for de-extinction in general.

Firstly, when considering de-extinction genome engineering, what types of loci are we expecting to be most relevant? There has been a great deal of success in identifying the causal variant underlying single gene disorders of large effect (often disease-causing loci, or extreme mutations). However, when considering the range of phenotypes that we would like to modify to create extinct species, we are often dealing with quantitative traits (for example, cold adaptation in the mammoth, aggregation and mating behaviour in the passenger pigeon, and so on). Such loci are typically identified via genetic mapping techniques, by either linkage-based QTL mapping, or genome-wide association studies in natural populations (GWAS). Such loci are far harder to identify down to the nucleotide level (i.e. to identify the actual causal mutation), and the confidence intervals of the detected loci can vary quite dramatically, as does the effect size (Hayes, 2013). In the case of a linkage-based QTL study, these are on average 10cM (Darvasi and Soller, 1994), so dependent on the size of the genome, but for example with mice 1cM ∼1Mb, whilst the average effect size of a QTL is typically ∼5 % of the total variation of the intercross (Flint, 2003; Lynch and Walsh, 1998). In contrast, GWAS studies have much smaller confidence intervals, but the effect size is commensurately also far smaller (typically less than a percent of the total variation) (Stringer et al., 2011). Even with the larger genomic regions that are now possible to transgenically modify/ rewrite (up to 1Mb, but typically far smaller regions are more viable currently in terms of cost), such regions could easily be far larger than the insert size. This means that it is necessary to fine map these regions so they are sufficiently small. In addition, it is also preferable if the loci identified have as large an effect as possible.

#### Genetical genomics

One method which can be used to try and identify and refine QTL regions as well as also identify the causal gene for a QTL are genetical genomics techniques. These techniques work by combining genotypes, gene expression, and organismal traits, all from the same individuals, as well as even such traits as methylation phenotypes and open chromatin regions (ATAC), to identify loci where a causal link can be statistically defined linking these different data (Höglund et al., 2020; Johnsson et al., 2016). This enables a statistical link to be drawn between genotypes that in turn modify gene expression, that in turn modify phenotype (Aten et al., 2008), or indeed between any of the other parameters used (so genotype – methylation – gene expression, for example (Höglund, Henriksen, Fogelholm, Churcher, Guerrero-Bosagna, Martinez Barrio, Johnsson, Jensen and Wright, 2020)). This genetical genomics approach can be applied to a range of data, for example this technique has been applied to identify causal genes arising from domestication in the chicken that affect anxiety behaviour (Fogelholm et al., 2019; Johnsson et al., 2018a, 2016), bone allocation (Johnsson et al., 2015a, 2015b), weight (Johnsson, et al., 2018b) and comb mass (Johnsson et al., 2012, 2014). However, even with these tools, causal genes, rather than the specific causal polymorphism, have been identified due to the use of bulk transcriptomic analysis and the limitations of resolution associated with linkage mapping. What this means is that whilst the gene that may be causal to a phenotype can be identified, the exact location of the actual polymorphism that causes this gene expression change remains uncertain.

One of the biggest limitations in ascertaining causation using expression analyses relates to how gene expression is typically measured, with many thousands of cells averaged for one sample. This often results in a poor measure of gene expression, especially if cell types have very different expression patterns. In contrast, single-cell techniques measure gene expression on a per-cell basis, with up to thousands of cells measured per sample. These have the potential to revolutionize causality mapping, and yet no development of this has yet occurred. Single cell networks are not currently used in this manner, but this has been identified as an area of promise (Tanay and Regev, 2017), particularly how gene regulation and genomic polymorphisms affect cell differentiation and tissue formation in a coordinated and flexible fashion. Currently “pseudo-time” is one method whereby cells are essentially ordered along a gradient to try and ascertain which genes are causal to other genes (Qiu et al., 2020). Although interesting, this does not address the more fundamental question of actual genotype-to-gene regulation. The most recent advances in single-cell techniques allow for the capture of gene expression profiles and open chromatin (ATAC) regions simultaneously, on an individual cell basis (using the 10X Multiome chip). These provide the perfect platform for causality analysis as many thousands of cells are available per individual. The next major advantage comes from the combined use of ATAC and gene expression data. ATAC data identifies the regions in the specific cell that are currently open and hence could contain the regulatory elements (Grandi et al., 2022). This can greatly refine the causality analysis, as these ATAC regions are far smaller and can reduce causal regions substantially. This offers the opportunity to assess how genotype affects open chromatin regions (ATAC-qtl) (Mu et al., 2026), and how these in turn affect gene expression, using precise cell-specific data. By orienting the relationship between open chromatin and target gene expression, causal analysis can be used as a fine-mapping method, identifying the promoters, enhancers and other chromatin elements that drive changes in gene expression.

To further expand on this, one limitation that can be potentially turned to an advantage with single cell techniques is the low sample size in terms of numbers of actual individual samples used. Whereas modern eQTL/ eGWAS have sample sizes of hundreds, or even thousands of individuals (Guan et al., 2025), the prohibitive cost of single cell technologies make such sample sizes extremely expensive. However, though the sample sizes are small, it is possible to leverage the large number of cells that are represented in the data. Typically with single cell QTL analysis, once cell clusters have been identified, expression is averaged over all cells for each individual in each cluster (so in essence it is cell-type specific bulk RNA analyses) (van der Wijst et al., 2020). However, this reduces data quite severely and doesn’t make optimal use of the high number of individual transcriptomes and ATACseq genomes that are generated. One possibility would be to instead include all cell transcriptomes, and then nest these within individual in a hierarchical model. This would then leverage the full data generated, and in particular for causation analysis (ascertaining causation from the SNP to open chromatin region to gene expression), should increase power, particularly if ATAC- and expression-QTL are available for the same cells.

#### The chicken as a model

The chicken would make an excellent model for such genetic alterations. Firstly, birds do not currently have the possibility to use the cloning strategy for de-extinction (Shapiro, 2017), meaning that for birds and reptiles, genome modification is the only real path for de-extinction. The chicken is the avian model with the greatest number of genetic tools (Dequéant and Pourquié, 2005; Zhou et al., 2025). It possesses a compact genome (∼1Gb in length) and a low number of repeats (Smith et al., 2023), reducing the sizes required for genomic rewrites and edits. In addition, it is possible to leverage the extreme phenotypic variation created by domestication, to compare with the original wild-type progenitor, the Red Jungle fowl . This gives a ready-made comparison, and such is the extreme variation between wild and domestic chickens, means that the magnitude is similar to between-, rather than within-species variation (Jensen and Wright, 2022; Wright et al., 2010). Therefore, a logical first test of whether such extreme phenotypic modification can be brought about using transgenic gene editing technology is to modify a domestic chicken to appear phenotypically wild, or vice versa.

In summary, this small review highlights some of the problems and possibilities of de-extinction, but proposes the use of a genetical genomics technique in combination with single cell technology to test some of the assumptions of phenotypic modification using transgenic technology, not only for de-extinction but for transgenic modification in general. The chicken would make the perfect model for such a project, in particular through leveraging the large phenotypic changes that exist between wild and domestic breeds.

Collectively, these contributions illustrate how the ChickenGTEx framework connects foundational regulatory mapping with sex-specific analyses and applied breeding strategies, forming a coherent platform for precision poultry genomics.

## Conclusions

The convergence of multi-tissue regulatory genomics, sex-informed molecular mapping, and integrative omics analyses is reshaping our understanding of complex trait architecture in chickens. Across growth, reproduction, metabolic efficiency, and extended laying performance, these studies collectively demonstrate that quantitative phenotypic variation is governed by coordinated regulatory networks rather than isolated genetic loci. Tissue-specific gene regulation, sex-dependent genetic effects, structural variation, and host–microbiome interactions together form a multilayered framework that links genotype to phenotype. By resolving this regulatory architecture, we move beyond association signals toward mechanistic insight.

A central implication of this work is the identification of loci that modifies large-scale quantitative phenotypic variation. Multi-omics integration enables prioritization of causal genes and regulatory variants with demonstrable biological function, thereby refining the targets available for molecular breeding. In addition to enhancing genomic selection strategies, these discoveries provide a rational foundation for precise genome modification through transgenic and gene-editing technologies. Targeted manipulation of validated regulatory loci offers the potential to fine-tune metabolic allocation, reproductive persistence, disease resilience, and production efficiency in ways that were previously unattainable using traditional selection alone.

Together, these advances signal a transition from descriptive genomics to predictive and actionable genomics in poultry science. As regulatory atlases expand and causal inference models mature, integrating functional annotation with genomic prediction and precision editing will accelerate sustainable genetic improvement. Such approaches will be critical for meeting the growing global demand for efficient, resilient, and environmentally responsible poultry production systems in the decades ahead.

## CRediT authorship contribution statement

**Lingzhao Fang:** Conceptualization, Writing – original draft, Writing – review & editing. **Haihan Zhang:** Writing – original draft, Writing – review & editing. **Congjiao Sun:** Writing – original draft, Writing – review & editing. **Xiangxiao Hu:** Writing – original draft, Writing – review & editing. **Dominic Wright:** Writing – original draft, Writing – review & editing. **Huaijun Zhou:** Conceptualization, Resources, Writing – original draft, Writing – review & editing.

## Disclosures

The authors declare that they have no known competing financial interests or personal relationships that could have appeared to influence the work reported in this paper.
